# Patient*innenzentrierte perioperative Versorgung: perioperative Prozessqualität, Effektivität von Schmerztherapie und Mobilisationsfortschritt nach Implementation eines Maßnahmenbündels bei Knietotalendoprothese

**DOI:** 10.1007/s00101-020-00874-8

**Published:** 2020-10-26

**Authors:** J. Spielberger, F. Heid, I. Schmidtmann, P. Drees, U. Betz, W. Schwaderlapp, G. Pestel

**Affiliations:** 1 Klinik für Anästhesiologie, Universitätsmedizin Mainz, Langenbeckstr. 1, 55131 Mainz, Deutschland; 2 Institut für Medizinische Biometrie, Epidemiologie und Informatik, Universitätsmedizin Mainz, Mainz, Deutschland; 3 Zentrum für Orthopädie und Unfallchirurgie, Universitätsmedizin Mainz, Mainz, Deutschland; 4 Institut für Physikalische Therapie, Prävention und Rehabilitation, Universitätsmedizin Mainz, Mainz, Deutschland

**Keywords:** Behandlungspfad, Heilverlauf, Patient*innenzentrierte perioperative Versorgung, PPV, Treatment bundle, Perioperative surgical home, Patient-centered outcomes, PSH

## Abstract

**Hintergrund:**

In den USA wurde das Konzept des „perioperative surgical home“ initialisiert, in dem ein teamorientiertes Vorgehen einen umfassenderen und zügigeren Heilverlauf erzielen soll.

**Fragestellung:**

Evaluation des Effekts eines interdisziplinären Maßnahmenbündels (patient*innenzentrierte perioperative Versorgung, PPV) auf Aspekte der Prozessqualität unter deutschen Rahmenbedingungen.

**Material und Methoden:**

Nach Einführung des PPV-Maßnahmenbündels (1. Patient*innenseminar, 2. spezifische Chirurgietechnik, 3. spezifische Anästhesietechnik, 4. Physiotherapiebeginn am Operationstag) wurden 34 Patient*innen mit elektiver Knietotalendoprothese prospektiv untersucht und mit „matched-pair“-Kontrollen verglichen. Endpunkte sind Dauer der Einleitungszeit (primär) und Krankenhausverweildauer, Ruhe- und Belastungsschmerz am 1. postoperativen Tag (numerische Analogskala), und Mobilisationsfortschritt (MBF) an den postoperativen Tagen 1, 3 und 6 (sekundär). Gruppenvergleiche wurden mit Wilcoxon-Mann-Whitney-Tests auf Nichtunterlegenheit durchgeführt. Im Fall von Nichtunterlegenheit wurde anschließend auf Überlegenheit getestet.

**Ergebnisse:**

Die Einleitungszeit in der PPV-Gruppe betrug im Median 13,5 min (Kontrollgruppe: 60 min, *p* < 0,0001), die Krankenhausverweildauer betrug in der PPV-Gruppe 8 Tage (Kontrollgruppe: 12 Tage, *p* < 0,0001). Am ersten postoperativen Tag betrug die mediane Ruheschmerzstärke in der PPV-Gruppe 30 (Kontrollgruppe: 20); die Belastungsschmerzstärke war in beiden Gruppen gleich (Median 40). Die Mobilisation der Patienten*innen der PPV-Gruppe gelang an den postoperativen Tagen 1, 3 und 6 besser (jeweils *p* < 0,0001).

**Schlussfolgerung:**

Das Konzept der patient*innenzentrierten perioperativen Versorgung (PPV) erscheint vielversprechend genug, um weitere klinische Studien zu rechtfertigen.

## Abstract (en)

**Background:**

The perioperative surgical home is a patient-centered, team-based model of care developed in the USA to coordinate diagnosis, treatment and follow-up; however, due to different healthcare systems, scientific findings in the USA cannot be simply transferred to Germany.

**Objective:**

This preliminary study was carried out to evaluate the effects of a new interdisciplinary treatment bundle (patient-centered perioperative vigilance, PPV) in a German university hospital.

**Material and methods:**

After IRB approval and written informed consent, 34 patients (PPV group) undergoing elective endoprosthetic surgery were enrolled after introduction of the PPV bundle (1. preoperative patient education, 2. specific surgical technique, 3. specific anesthesia technique, 4. start of mobilization on day of operation) and compared to historic matched pairs (HMP) for age cohort, ASA-PS, body mass index, and sex. We hypothesized that PPV shortens induction time (primary outcome). Secondary outcomes were length of hospital stay (LOS), resting pain and pain with movement on postoperative day 1 and mobilization progress on postoperative days 1, 3 and 6. Groups were compared with Wilcoxon-Mann-Whitney test for noninferiority. In the case of noninferiority, a Wilcoxon–Whitney–Mann test for superiority was additionally applied.

**Results:**

The median anesthesia induction time was 13.5 min for PPV and 60 min for HMP (*p* < 0.0001). The LOS was 8 days for PPV and 12 days for HMP (*p* < 0.0001). Resting pain on postoperative day 1 was 20 for PPV (30 for HMP). Pain with movement was identical (median 40). Mobilization progress was better for PPV on days 1, 3 and 6 (*p* < 0.0001 for each day).

**Conclusion:**

The concept of patient-centered perioperative vigilance (PPV) shortens induction time and hospital length of stay. Mobilization improves with PPV on day 1. Higher pain scores in PPV seem to be clinically insignificant, which warrants further study.

## Einleitung

2017 wurden in Deutschland 16,8 Mio. Operationen durchgeführt [24], 57 % der insgesamt 19,9 Mio. stationär versorgten Patient*innen hatten eine Krankenhausverweildauer von mehr als 3 Tagen [25]. Bei einer Krankenhausverweildauer von 10 Tagen (Median) wurden im Jahr 2017 in Deutschland 188.124 Knietotalendoprothesen implantiert [13]. Die Krankenhausverweildauer in Deutschland ist im OECD-Vergleich von 29 Staaten nach Japan am höchsten [19]. Es gibt Hinweise, dass die Patientenversorgung in häuslicher Umgebung der Versorgung im Krankenhaus gleichwertig sein könnte, bei größerer Patientenzufriedenheit und geringeren Kosten [21].

Zur Senkung der Krankenhausverweildauer durch Koordinierung von Diagnostik, Therapie und Nachsorge im Rahmen operativer Eingriffe wurde in den USA das Konzept des „perioperative surgical home“ [1] initialisiert. Dieses Konzept basiert auf der Vorstellung, dass die lange Zeit übliche vertikale Strukturierung des Gesundheitswesens in ein teamorientiertes Vorgehen transformiert werden sollte [15], in dem bei patientenspezifischer Anwendung von „evidence-based medicine“ ein umfassender und zügiger Heilverlauf angestrebt wird, zum Wohle der Patient*innen, der Leistungserbringer und der Gesellschaft [28]. Zentraler Leitgedanke des Konzepts ist die interdisziplinäre Kooperation aller beteiligten Berufsgruppen mit dem Ziel einer optimalen Patientenversorgung bei umsichtiger Nutzung von Zeit und Ressourcen [5]. Dabei arbeitet ärztliches und nichtärztliches Personal gemeinsam an der Aufgabe, für die jeweilige Institution ein Konzept zu erarbeiten, in dem informierte und aufgeklärte Patient*innen nach Regeln gesicherten Wissens und guter klinischer Praxis mittels ineinandergreifender Behandlungsschritte von der präoperativen Entscheidungsfindung bis zur postoperativen Nachbetreuung kontinuierlich anhand konsensual entwickelter Schulungs- und Behandlungsprotokolle versorgt werden [15, 27].

Da im deutschen Gesundheitswesen ein solches Vorgehen bislang nicht evaluiert ist, werden mit vorliegender Studie Aspekte der perioperativen Prozessqualität am Beispiel der Anästhesieeinleitungszeit, der suffizienten Schmerztherapie und des zügigen Mobilisationsfortschrittes vor und nach Implementation eines Maßnahmenbündels an der Universitätsmedizin Mainz (präoperative Patientenschulung, modifiziertes chirurgisches und anästhesiologisches Vorgehen, nichtinvasive Schmerztherapie, frühzeitige Mobilisation) bei Knietotalendoprothese verglichen.

## Material und Untersuchungsmethoden

**Figure 1:**
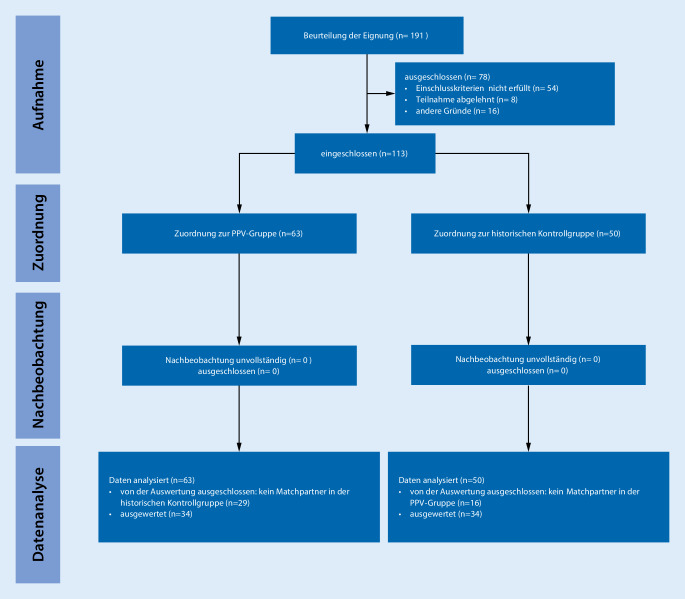

Nach schriftlicher Genehmigung des nach den STROBE-Leitlinien [30] erstellten Studienprotokolls durch die zuständige Ethikkommission (Ethikkommission der Landesärztekammer Rheinland-Pfalz, Vorsitzender: Univ.-Prof. Dr. med. habil. Dipl.-Ing. Stephan Letzel, Bearbeitungsnummer 837.280.18 (10599) vom 07.11.2016), ausführlicher Aufklärung und schriftlicher Einwilligung wurden 34 Patient*innen mit fortgeschrittener radiologisch nachgewiesener Arthrose des Kniegelenks nach ausreichender konservativer Therapie, deutlich eingeschränkter Lebensqualität und Indikation zur Implantation einer Knietotalendoprothese (Knie-TEP) nach Einführung des Maßnahmenbündels patient*innenzentrierte perioperative Versorgung (PPV) der Universitätsmedizin Mainz in einer prospektiven, monozentrischen Observationsstudie untersucht (PPV-Gruppe) und mit „matched-pair“-Kontrollen (Infobox 1) einer Vergleichspopulation (*n* = 34) vor Einführung des Maßnahmenbündels verglichen (Kontrollgruppe). Einen Überblick ergibt das Flussdiagramm in Abb. 1.

Da die Studie nicht prospektiv randomisiert, sondern als prospektive Beobachtungsstudie mit einem historischen Kollektiv als Kontrollgruppe geplant war, wurde auf eine Registrierung bei einer öffentlichen Datenbank für klinische Studien verzichtet. Der primäre Endpunkt (Dauer der Anästhesieeinleitung), aufgrund dessen die Abschätzung der Stichprobengröße erfolgte, war vor Studienbeginn in dem der Ethikkommission zur Beschlussfassung vorgelegten Studienprotokoll schriftlich niedergelegt worden.

Geplante Regionalanästhesie, Dauermedikation mit Opioiden, ASA-PS-Klassifikation >3, kognitive und/oder kommunikative Defizite, die nach Meinung des Prüfarztes den Erfolg einer adäquaten Studienaufklärung ungewiss erschienen ließen, sowie Teilnahme an pharmakologischen Studien innerhalb der letzten 28 Tage galten als Ausschlusskriterien.

### Anteile des Maßnahmenbündels

Bei einem obligaten Patient*innenseminar [6] 2 bis 4 Wochen vor dem geplanten Operationstermin werden Patient*in und Coach (eine ausgewählte Vertrauensperson) der perioperative Ablauf und Details der Operation, der Anästhesie und der Schmerztherapie sowie der Physiotherapie und daraus resultierender Entlassungskriterien (Ankleiden ohne Hilfe, selbstständiges Ein- und Aussteigen aus dem Bett, selbstständiges Hinsetzen auf und Aufstehen von einem Stuhl oder von der Toilette, persönliche Hygiene ohne Hilfe, Mobilisierung mit Unterarmgehhilfen, 70 m Gangdistanz mit Unterarmgehhilfen, ausreichende Schmerzbehandlung mit Schmerzstärke <50 auf einer numerischen Analogskala (0: keine Schmerzen; 100: stärkster vorstellbarer Schmerz) bei Aktivität) durch Verantwortliche der jeweiligen Fachbereiche in einer offenen und wertschätzenden Atmosphäre dargestellt.

Die organisatorische Optimierung von präoperativen Abläufen (z. B. chirurgische und anästhesiologische Aufklärung am Tag des Patient*innenseminars) und zeitnaher Einbestellung der Patient*innen am Operationstag mit kurzer Nüchternheitsphase wurde akribisch geplant und durchgeführt.

Die Allgemeinanästhesie wurde mit Propofol, Sufentanil und Atracurium gewichtsadaptiert eingeleitet und mit Sufentanil und Sevofluran aufrechterhalten. Auf Nervenblockaden und Katheterverfahren zur Analgesie wurde verzichtet. Stattdessen wurde eine opioidsparende multimodale Analgesiestrategie mit oraler Gabe von Dexamethason, Etoricoxib und Oxycodon sowie zusätzlicher Etablierung einer intraoperativen lokalen Infiltrationsanalgesie (LIA, Anlage durch den Operateur) [7] verfolgt (200 ml Ropivacain 0,2 %, davon 150 ml + 1 mg Epinephrin periartikulär in sämtliche chirurgisch adressierten Gewebe sowie 50 ml ohne Epinephrin s.c.). Als Reservemedikation im Aufwachraum bei Schmerzdurchbruch wurde Piritramid vorgehalten. Tranexamsäure wurde bei Operationsbeginn (1 g i.v.) und nach Kapselverschluss (2 g i.a.) gegeben [8]. Hämoderivate wurden nach PBM-Kriterien [22] verabreicht.

Patient*innen der Kontrollgruppe erhielten vor Narkoseeinleitung sonographisch gesteuert einen N.-femoralis-Katheter. Zur postoperativen kontinuierlichen Katheteranalgesie wurde Ropivacain 0,2 % perineural infundiert (6–12 ml/h). Ergänzend wurden die Patient*innen mit einer i.v.-PCA (patientenkontrollierte Analgesie) versorgt (Piritramidbolus 3 mg, Sperrzeit 10 min, 8‑h-Maximum 30 mg, keine Basalrate). Begleitend wurde in Abhängigkeit von Vorerkrankungen Diclofenac, Paracetamol oder Metamizol verordnet. Hinsichtlich der Durchführung der Allgemeinanästhesie gab es keine Gruppenunterschiede.

Im Rahmen des Maßnahmenbündels wurde chirurgisch auf eine atraumatische Operationstechnik geachtet, mit Vermeidung von pneumatischer Blutsperre [12], minimaler Schnittführung mit Nutzung natürlicher Muskellücken, Verzicht auf Drainagen und intraoperativer Kontrolle des Operationserfolges mittels bildgebendem Verfahren.

Physiotherapeutisch waren alle Bewegungen und Körperhaltungen erlaubt [11]. Lagerungsmaterial und passive Bewegungsschienen wurden nicht eingesetzt. Im Mittelpunkt standen Maßnahmen zur Steigerung der individuellen Handlungsfähigkeit („empowerment“) und Motivation zu selbstständiger Aktivität, das Erarbeiten der Selbstständigkeit im Alltag, Angehörigenanleitung, Vermittlung von Eigenübungsprogrammen, Vorgaben strukturierter Aktivitätsprogramme und regelmäßiges Screening der Funktionsfähigkeit. Die Patient*innen verließen bereits am Operationstag aktiv das Bett und wurden ermuntert, Aktivitätsangebote wie Bewegungsparcours und Outdoor-Gruppe in Anspruch zu nehmen.

### Endpunkte

Primärer Endpunkt war die Dauer der Einleitungszeit, welche im Rahmen der Anästhesiedokumentation erhoben wurde. Dieser Parameter wurde aufgrund der Erwartung des stärksten Effektes bei Einführung des PPV-Maßnahmenbündels gewählt. Als sekundäre Endpunkte wurden die Krankenhausverweildauer (LOS) sowie Ruhe- und Belastungsschmerz (RSZ, BSZ) am 1. postoperativen Tag gemessen. Zur Quantifizierung wurde eine numerische Analogskala (0: keine Schmerzen; 100: stärkster vorstellbarer Schmerz) verwendet. Als weiterer sekundärer Endpunkt wurde der Mobilisationsfortschritt (MBF) mithilfe einer Mobilisationsstufenskala von 0 bis 5 (0: keine Mobilisation, 1: Patient*in sitzt an der Bettkante, 2: Patient*in steht, 3: Patient*in geht auf Zimmerebene, 4: Patient*in geht auf Flurebene, 5: Patient*in steigt Treppen) an den postoperativen Tagen 1, 3 und 6 erhoben.

### Statistische Methoden

Ziel war es, die mittlere Einleitungszeit mit einem 95 %-Konfidenzintervall zu bestimmen, das eine 95 %-Überdeckungswahrscheinlichkeit hat und einen Bereich ±10 min um die beobachtete mittlere Einleitungszeit umfasst. Eine retrospektive Analyse früher dokumentierter Einleitungszeiten ergab annähernd normal verteilte Einleitungszeiten mit einer Standardabweichung von 23,6 min. Mit dieser Grundlage ergibt sich für die PPV-Gruppe ein Stichprobenumfang von *n* = 34.

Zum Vergleich der Patient*in der PPV-Gruppen mit den historischen Kontrollen wurden für die primären und sekundären Zielgrößen Wilcoxon-Mann-Whitney-Tests angewandt, da eine Normalverteilung nicht überall angenommen werden konnte. Zunächst wurde jeweils geprüft, ob gegenüber der historischen Kontrolle Nichtunterlegenheit nachgewiesen werden konnte. Die Nullhypothese besagte jeweils, dass $P\left(X>Y\right)+\frac{1}{2} P\left(X=Y\right)\leq \frac{1}{2}-\varepsilon$, mit Nichtunterlegenheitsschranke $\varepsilon =0,1$. Dabei steht X für den in der Vergleichsgruppe beobachteten Wert der Zielgröße, Y für den in der PPV-Gruppe beobachteten Wert, und höhere Werte werden als ungünstig angesehen. Wenn dies möglich war, wurde anschließend noch auf Überlegenheit geprüft. Alle Tests wurden einseitig zum 2,5 %-Niveau durchgeführt. Eine Korrektur für multiples Testen wurde nicht vorgenommen; es wird also nur das lokale Signifikanzniveau eingehalten.

Die Analysen wurden mit SAS 9.4 (SAS Institute, Cary, NC, USA) und R 3.5.0 (R Foundation for Statistical Computing, Wien, Österreich) durchgeführt.

## Ergebnisse

34 Patient*innen wurden prospektiv nach Einführung des perioperativen Maßnahmenbündels „patient*innenzentrierte perioperative Versorgung“ untersucht (PPV-Gruppe) und mit 34 Patient*innen vor Einführung des Maßnahmenbündels nach „matched-pair“-Kriterien verglichen. Eine Übersicht der demografischen Daten und des Matching zeigt Tab. 1.

**Table 1:** 

	Patientenkollektiv					
	PPV-Gruppe		Kontrollgruppe			
	*n*	MW ± SD	*n*	MW ± SD		
Alter (Jahre)	34	67,5 ± 11,1	34	66,9 ± 12,6		
	Patientenkollektiv				Total	
	PPV-Gruppe		Kontrollgruppe			
	*n*	%	*n*	%	*n*	%
Geschlecht						
– Weiblich	18	53	18	53	36	53
– Männlich	16	47	16	47	32	47
Gewichtsklasse						
– BMI <25 kg/m^2^	4	12	4	12	8	12
– BMI 25–30	12	35	12	35	24	35
– BMI >30	18	53	18	53	36	53
ASA-PS						
– 1	2	6	2	6	4	6
– 2	19	56	19	56	38	56
– 3	13	38	13	38	26	38

*ASA-PS* American Society of Anesthesiologists Physical Score, *BMI* Body-Mass-Index, *MW* Mittelwert, *PPV* patient*innenzentrierte perioperative Versorgung, *SD* Standardabweichung

**Figure 2:**
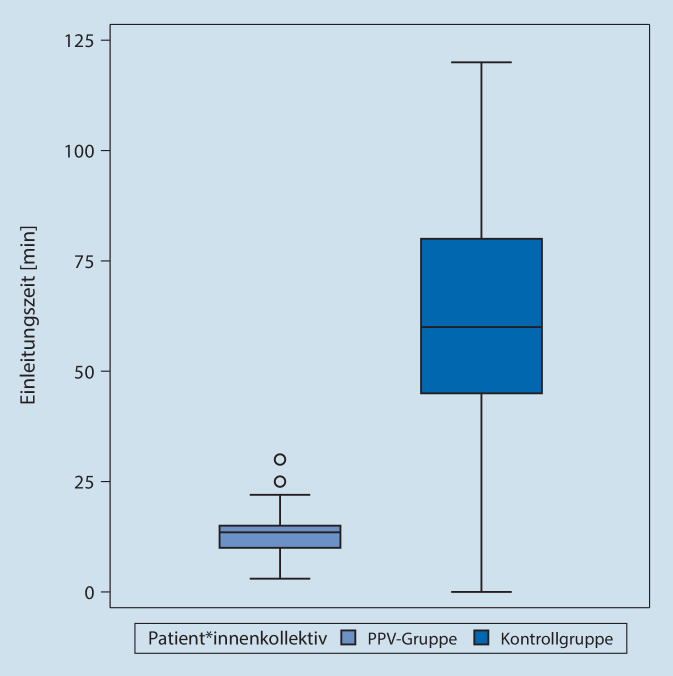

Bei den historischen Kontrollen betrugt die mediane Einleitungszeit 60 min (Mittelwert = 61,1 min), im PPV-Kollektiv betrugen Median und Mittelwert jeweils 13,5 min (Abb. 2). Das 95 %-Konfidenzintervall für den Mittelwert ergab [12,0 min; 15,4 min]. Im Vergleich mit den historischen Kontrollen ergab sich eine signifikant kürzere Einleitungszeit (*p* < 0,0001).

**Figure 3:**
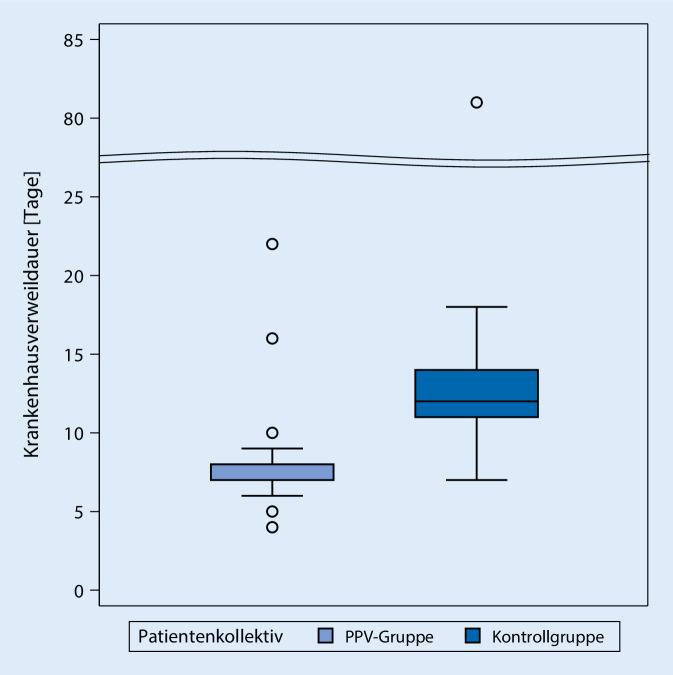

Während die mediane Krankenhausverweildauer (Abb. 3) der historischen Kontrollen bei 12 Tagen (IQR 11 bis 14 Tage) lag, war sie bei den PPV-Patient*innen mit 8 Tagen im Median (IQR 7 bis 8 Tage) signifikant kürzer (*p* < 0,0001).

**Figure 4:**
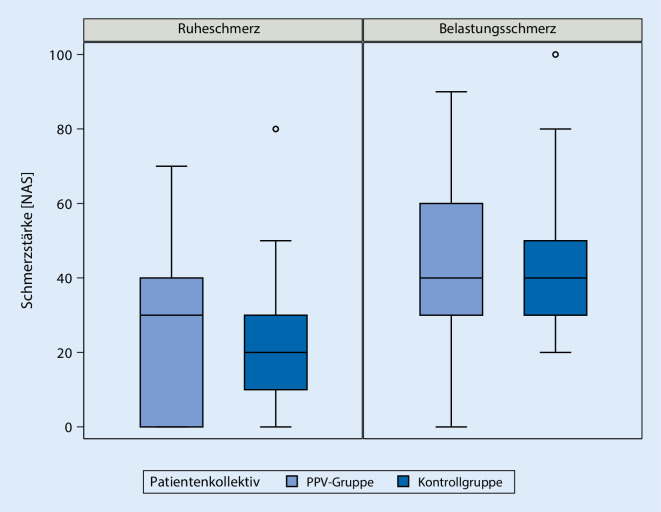

Am ersten postoperativen Tag wurde der von den Patient*innen im PPV-Kollektiv auf einer Skala von 0 bis 100 eine mediane Ruheschmerzstärke von 30 (IQR 2,5–40) angegeben, während die Patient*innen aus der historischen Kontrolle im Median 20 (IQR 10–30) angaben. Die Belastungsschmerzstärke war im Median in beiden Gruppen 40 (IQR PPV 30–60, Kontrollen 30–50). Weder für Ruheschmerz noch für Belastungsschmerz konnte bei PPV Nichtunterlegenheit nachgewiesen werden (Ruheschmerz: *p* = 0,1711, Belastungsschmerz *p* = 0,2512) (Abb. 4).

**Figure 5:**
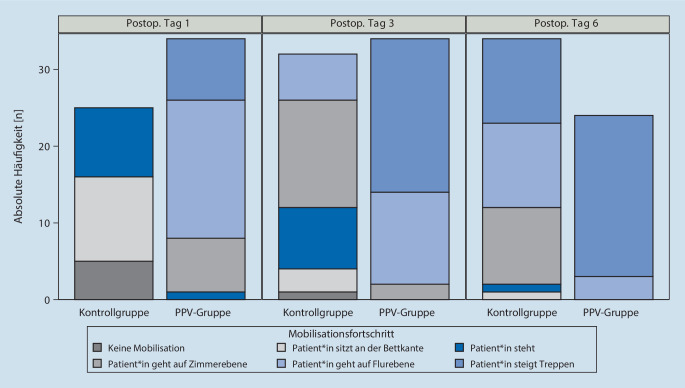

Eine Übersicht über den Mobilisationsfortschritt gibt Abb. 5: Am ersten Tag unterschied sich die Mobilisation sehr deutlich zwischen den beiden Patientengruppen (*p* < 0,0001). Während von den Kontrollen nur 9 überhaupt das Bett verließen und standen, verließen alle PPV-Patient*innen das Bett. 18  gingen auf Flurebene, 8 stiegen Treppen. Auch am dritten Tag unterschieden sich die beiden Gruppen noch sehr deutlich in der Mobilisation (*p* < 0,0001). Treppensteigen gelang in der PPV-Gruppe 20 Patient*innen (Kontrollgruppe 0). Der Unterschied in der Mobilisation bestand auch noch am sechsten Tag (*p* < 0,0001). Zwar konnten in der Kontrollgruppe zu diesem Zeitpunkt bis auf 2 alle gehen, jedoch nur je 11 auf dem Flur oder bereits Treppen steigen. In der PPV-Gruppe konnten 2 Patient*innen auf dem Flur gehen; die übrigen verbliebenen 21 dokumentierten Patient*innen konnten Treppen steigen; 8 waren zu diesem Zeitpunkt bereits entlassen.

## Diskussion

Patient*innenzentrierte perioperative Versorgung (PPV) ist ein interdisziplinäres Maßnahmenbündel der Universitätsmedizin Mainz bei Knietotalendoprothese. Es besteht aus obligater präoperativer Patientenschulung, spezifischer chirurgischer Technik, spezifischer Anästhesietechnik mit Ersatz von Katheteranalgesie durch eine Kombination aus lokaler intraoperativer Analgesie mit opioidsparender multimodaler Analgesie und Beginn der präoperativ eingeübten Physiotherapie (Gehen an Unterarmgehstützen sowie ein Eigenübungsprogramm zur Thromboseprophylaxe, Aktivierung und Dehnung der Beinmuskulatur sowie der Mobilisation der Kniegelenke) noch am Operationstag. PPV bewirkt neben einer deutlich verkürzten Anästhesieeinleitungszeit eine Reduktion der Krankenhausverweildauer sowie einen verbesserten postoperativen Mobilisationsfortschritt unter suffizienter Schmerztherapie.

Die Implementation des Maßnahmenbündels (u. a. Ersatz von Katheteranalgesie durch eine Kombination aus lokaler Intraoperativer Analgesie mit opioidsparender multimodaler Analgesie) führte zu einer Reduktion der Einleitungszeit von im Median 60 min auf 13,5 min. Eine Zeiteinsparung von mehr als 45 min im Rahmen der Einleitung gestattet nicht nur einen zügigeren Operationsbeginn im Tagesablauf, sondern eröffnet auch Gestaltungsräume für die Verwendung von Anästhesiepersonal durch Verminderung der Intensität bei der Personalbindung. Im US-amerikanischen Schrifttum wurde eine Effizienzsteigerung der perioperativen Prozesse als Möglichkeit postuliert, eine „durchschlagende Innovation“ [10] im Gesundheitswesen mit dem Ziel der einfacheren, zugänglicheren und preisgünstigeren Gesundheitsversorgung zu erreichen. In einer monozentrischen Observationsstudie, bei der ein Zweijahreszeitraum vor Einführung eines perioperativen Maßnahmenbündels in der Endoprothetik mit einem Zweijahreszeitraum nach Einführung des Maßnahmenbündels verglichen worden war, zeigte sich eine signifikante Zunahme des Anteils von Fällen mit pünktlichem Operationsbeginn [29]. In einer Übersichtsarbeit zu den Auswirkungen der Einführung von perioperativen Maßnahmenbündeln auf Prozessqualität und Kosteneffizienz [16] konnte gezeigt werden, dass in der großen Mehrzahl der Programme Verbesserungen erzielt werden konnten (77 % bei Programmen in den USA, 88 % bei Programmen außerhalb der USA).

Die Implementation des Maßnahmenbündels bei elektiver Knietotalendoprothese führte zu einer Reduktion der Krankenhausverweildauer von im Median 12 (11 bis 14) Tagen auf 8 (7 bis 8) Tage. Obwohl der Parameter „Krankenhausverweildauer“ nicht die primäre Zielsetzung und den Endpunkt vorliegender Studie darstellt, verdient die hochsignifikante und für Patient*innen, Behandelnde und das Gesundheitswesen insgesamt bedeutsame Verminderung des Aufenthalts im Krankenhaus (Median) um 4 Tage entsprechende Aufmerksamkeit.

In 2 US-amerikanischen Studien [18, 29] konnte durch die Einführung eines spezifischen Behandlungsprotokolls keine Veränderung der Krankenhausverweildauer erreicht werden. Allerdings wurde in keiner der beiden Studien die Einführung eines therapeutischen Maßnahmenbündels evaluiert. Generell ist in den USA gemäß einer landesweiten Erhebung an über 1 Mio. Patient*innen die Krankenhausverweildauer bei endoprothetischen Eingriffen mit 3 Tagen (Median) sehr kurz, bemerkenswert erscheint dabei aber, dass eine Entlassung aus dem Krankenhaus (Verlegung nach Hause) nur in 47,1 % der Fälle beschrieben wird [4].

Einer ausführlichen präoperativen Aufklärung und Schulung bei Patient*innen, die sich einer Knietotalendoprothesenoperation unterziehen, wird ein hoher Stellenwert zur positiven Beeinflussung des Heilverlaufs eingeräumt [16]. In einer nordenglischen Studie an insgesamt 472 Patient*innen konnte durch ein vor der Operation interdisziplinär durchgeführtes Programm zu Aufklärung und Schulung eine Verminderung der Krankenhausverweildauer (Median) von 7 Tagen auf 5 Tage erreicht werden [14]. Möglicherweise trägt die in unserem Maßnahmenbündel integrierte obligate Patientenschulung zur beobachteten Verminderung der Krankenhausverweildauer bei.

Nach Implementation des Maßnahmenbündels bei elektiven Knietotalendoprothesen wurde bei betroffenen Patient*innen am ersten postoperativen Tag im Median eine Schmerzintensität von 30 (2,5–40) auf einer numerischen Analogskala von 0–100 gemessen, gegenüber einer Schmerzintensität von 20 (10–30) vor Einführung des Maßnahmenbündels. Hinsichtlich des Belastungsschmerzes am ersten postoperativen Tag wurden im Median Werte von 40 (30–60) nach Einführung des Maßnahmenbündels erhoben, gegenüber Werten von 40 (30–50) vor Einführung des Maßnahmenbündels. Zwar bewegt sich anscheinend die erlebte Schmerzintensität der Patient*innen nach Einführung des Maßnahmenbündels noch im tolerablen Bereich; ein statistischer Nachweis der Nichtunterlegenheit des schmerztherapeutischen Bemühens nach Einführung des Maßnahmenbündels gelang jedoch nicht.

Drei US-amerikanische deskriptive Beobachtungsstudien [3, 9, 26] nach Einführung eines „Perioperative-surgical-home“-Konzepts berichten über Schmerzintensität am 1. postoperativen Tag; eine Differenzierung zwischen Ruheschmerz und Belastungsschmerz wurde nicht vorgenommen. Die in vorliegender Untersuchung beobachtete postoperative Schmerzintensität ist somit vergleichbar mit den Befunden der US-amerikanischen Studien. Allerdings ist aufgrund des Vergleichs mit der Kontrollgruppe (vor Einführung des Maßnahmenbündels) zukünftig der Fragestellung Vermeidung von Schmerzen/Schmerztherapie bei Patient*innen mit Knietotalendoprothese unter Durchführung des Maßnahmenbündels besondere Aufmerksamkeit zu widmen. Dabei scheint die durchgeführte Anästhesietechnik selbst von geringerer Bedeutung zu sein als früher angenommen. Insbesondere eine Regionalanalgesie mit immobilisierenden Katheterverfahren zur postoperativen Schmerztherapie ist anderen Behandlungsoptionen nicht überlegen [20]. Konsequenterweise verzichten aktuelle Empfehlungen [23, 31] auf eine Festlegung hinsichtlich eines spezifischen Anästhesieverfahrens, solange allgemeine Therapieziele (standardisiertes anästhesiologisches Vorgehen unter Erhalt von Normovolämie, Normothermie und Normoglykämie, Vermeidung von PONV und postoperativem Schmerz durch multimodale Analgesie mit dem Ziel der frühen Mobilisierbarkeit) erreicht werden.

Vor Einführung des Maßnahmenbündels waren bei elektiven Knietotalendoprothesen ein Fünftel der Patient*innen am ersten postoperativen Tag bettlägerig und ein Drittel gerade in den Stand zu mobilisieren. Gründe waren die allgemein akzeptierte Vorstellung, dass Patient*innen nach der Operation zunächst ruhen sollten; Übelkeit, Schmerz und Schwindel waren schnell akzeptierte Gründe gegen aktive Mobilisierung, d.h., Mobilität wurde weniger unterstützt und eingefordert. Zudem bestanden zahlreiche Mobilitätsbarrieren (Lagerungsschienen, Blasenkatheter, Femoraliskatheter, i.v.-Zugänge, Bewegungsverbote). Die Einführung des Maßnahmenbündels bewirkte eine hochsignifikante Steigerung der postoperativen Mobilisierbarkeit. Der Erfolg der Frühmobilisierung in vorliegender Untersuchung erscheint vergleichbar mit einer amerikanischen Studie an 146 Patient*innen nach endoprothetischem Eingriff [9], bei der von 100 %iger Mobilisierung in den Stand innerhalb von 24 h nach Operation berichtet wird. Inwieweit die Mobilisierbarkeit nach Kniegelenkersatz durch Maßnahmen der „Prähabilitation“ (d. h. Maßnahmen VOR einem operativen Eingriff, mit dem Ziel der präoperativen Stärkung zur beschleunigten postoperativen Erholung) gesteigert werden kann, ist augenblicklich Gegenstand intensiver klinischer Forschung. Während eine frühere Metaanalyse bei Patient*innen mit endoprothetischem Gelenkersatz einen nur bescheidenen Effekt der Prähabilitation auf den Heilverlauf vermutete [32], konnte eine Metaanalyse bei solchen mit Kniegelenkersatz Verbesserungen der Mobilisierbarkeit mit Auswirkungen auf die Krankenhausverweildauer nachweisen [17]. Eine zusätzliche Steigerung des Effektes von Prähabilitationsmaßnahmen könnte zukünftig durch eine patientenspezifische Therapie im Sinne von „personalisierter Medizin“ erreicht werden [2].

Die vorliegende Arbeit beinhaltet mehrere Limitationen: Erstens handelt es sich nicht um eine randomisierte prospektive Studie. Zwar wurden Patient*innen nach Einführung des Maßnahmenbündels prospektiv untersucht, bei der Vergleichsgruppe handelt es sich aber über eine retrospektiv ausgewählte Kontrolle nach „matched-pair“-Kriterien. Ein solcher Vergleich ist methodenbedingt mit Unzulänglichkeiten behaftet, bietet aber zumindest Anhaltspunkte, die in reinen Beobachtungsstudien ohne Vergleichsgruppe nicht möglich sind. Zweitens erscheint der Mobilisationsfortschritt in der prospektiven Gruppe gegenüber der historischen Kontrolle augenfällig und besitzt statistische Signifikanz. Aufgrund der unterschiedlichen Fallzahlen an den postoperativen Tagen 1, 3 und 6 wären definitive Schlussfolgerungen derzeit allerdings verfrüht. Drittens kann die Generalisierbarkeit der gefundenen Ergebnisse hinterfragt werden, da es sich bei der Universitätsmedizin Mainz um ein Krankenhaus der Maximalversorgung handelt, mit entsprechenden Auswirkungen auf das Patientenkollektiv und klinikinterne Handlungsabläufe. Schließlich haben alle Schlussfolgerungen aufgrund der insgesamt geringen Gruppengrößen eher deskriptiven als beweisenden Charakter.

Zusammenfassend wurden in einer prospektiv angelegten Beobachtungsstudie an 34 Patient*innen mit elektiver Knietotalendoprothese nach Einführung eines interdisziplinären Maßnahmenbündels Effekte der Prozessqualität untersucht und einem „matched-pair„-Vergleich (Alter, Geschlecht, BMI-Gruppe, ASA-PS-Klasse) einer historischen Kohorte unterzogen. Dabei zeigte sich eine deutliche Verkürzung von Anästhesieeinleitungszeit und Krankenhausverweildauer bei verbesserter Mobilisierbarkeit. Die Ergebnisse weisen darauf hin, dass die Einführung und konsequente Anwendung eines interdisziplinar vereinbarten Maßnahmenbündels im Sinne des US-amerikanischen „perioperative surgical home“-Konzepts auch unter den Bedingungen des deutschen Gesundheitswesens vorteilhafte Effekte für Patient*innen, Behandelnde und Gesellschaft haben kann. Vor diesem Hintergrund haben Einrichtungen der Universitätsmedizin Mainz und Kooperationspartner das durch den Innovationsfonds beim Gemeinsamen Bundesausschuss geförderte PROMISE-Projekt mit dem Ziel der Entwicklung eines Best-Practice-Leitfadens für einen optimierten Gesamtversorgungsprozess bei Gelenkerkrankungen gestartet. Die Autor*innen schlagen als deutschen Begriff „patient*innenzentrierte perioperative Versorgung (PPV)“ vor.

## Kernaussagen

- Das interdisziplinäre therapeutische Maßnahmenbündel „patient*innenzentrierte perioperative Versorgung, PPV“ der Universitätsmedizin Mainz führt für die Patient*innen zu einem zügigen Heilverlauf.
- „Patient*innenzentrierte perioperative Versorgung, PPV“ ist ein Modell für einen optimierten Gesamtversorgungsprozess bei Patient*innen mit Knietotalendoprothese.
- „Patient*innenzentrierte perioperative Versorgung, PPV“ beschleunigt die Anästhesieeinleitung und den Mobilitätsfortschritt und verkürzt die Krankenhausverweildauer.
- Die erlebte Schmerzintensität der Patient*innen nach Einführung von „patient*innenzentrierter perioperativer Versorgung, PPV“ bleibt im tolerablen Bereich.

### Infobox 2 Maßnahmenbündel „Patient*innenzentrierte perioperative Versorgung, PPV“ der Universitätsmedizin Mainz bei Knietotalendoprothese

- Enge interdisziplinäre Abstimmung in regelmäßigen Treffen der Steuergruppe
- Präoperatives Patient*innenseminar mit Patientencoach
- Organisatorische Optimierung von präoperativen Abläufen
- Balancierte Allgemeinanästhesie ohne Katheterverfahren
- Opioidsparende multimodale Analgesie
- Tranexamsäure intraoperativ i.v. und lokal
- Hämoderivate nach PBM-Kriterien
- Atraumatische Operationstechnik und intraoperative Kontrolle des Operationserfolges
- Physiotherapeutische Maßnahmen zur Steigerung der individuellen Handlungsfähigkeit mit Aktivitätsangeboten wie Bewegungsparcours und Outdoor-Gruppe

### Infobox 1 „matched-pair“Kriterien

1. Alterskohorte (Fünfjahreszeitraum)
2. Geschlecht männlich *vs.* Geschlecht weiblich
3. BMI <25 kg/m^2^ *vs.* BMI 25–30 kg/m^2^ *vs.* BMI >30 kg/m^2^
4. ASA-PS 1 und 2 *vs.* ASA-PS 3

## Fazit für die Praxis

- Das Konzept „patient*innenzentrierte perioperative Versorgung, PPV“ ist ein interdisziplinärer Ansatz auf dem Fundament des Vertrauensgrundsatzes und der strikten Arbeitsteilung aller beteiligten Berufsgruppen mit dem Ziel des optimierten Heilverlaufs für die Patient*innen. Leitmotiv ist die Verzahnung aller patientenrelevanten Tätigkeiten. Die akribische Vorbereitung des Gesamtkonzeptes und dessen Weiterentwicklung im laufenden Betrieb erfordern von allen beteiligten Berufsgruppen konsensorientierte Innovations- und Dialogbereitschaft.
- Das Hinterfragen und Verwerfen vertrauter Automatismen zugunsten von evidenzbasiertem Handeln bedarf kritischer Reflexion und Überzeugungsarbeit, zunächst in den einzelnen Berufsgruppen, dann zwischen den einzelnen Berufsgruppen. Die Benennung von Irrwegen und Sackgassen ist unvermeidlich und unproblematisch, wenn notwendig gewordene Kurskorrekturen auf das gemeinsame Ziel des optimierten Heilverlaufs für die Patient*innen ausgerichtet sind.
- Eine präzise, verständliche und umfassende Patientenschulung ist für die aktive Mitarbeit der Patient*innen unerlässlich.
- Die Steuerung des Gesamtkonzepts, dessen Fortschreibung nach Überprüfen der Zielerreichung und die Aufnahme von Vorschlägen aus der Schwarmintelligenz aller Mitarbeitenden wird durch Verantwortliche, die in regelmäßigem Austausch miteinander stehen, bewerkstelligt.
